# Pre-treatment serum extracellular vesicle-enriched microbiome profiles and SSRI treatment response in obsessive-compulsive disorder: a prospective naturalistic cohort study

**DOI:** 10.3389/fpsyt.2026.1913728

**Published:** 2026-09-03

**Authors:** Jee In Kang, Chun Il Park, June-Ho Seo, Chil-sung Kang, Yoon-Keun Kim, Se Joo Kim

**Affiliations:** 1 Department of Psychiatry, Yonsei University College of Medicine, Seoul, Republic of Korea; 2 Institute of Behavioral Science in Medicine, Yonsei University College of Medicine, Seoul, Republic of Korea; 3 Department of Psychiatry, Yonsei University Wonju College of Medicine, Wonju, Kangwon, Republic of Korea; 4 MD Healthcare Inc., Seoul, Republic of Korea

**Keywords:** extracellular vesicles, microbiome, microbiota-brain interactions, OCD, prospective design, treatment response

## Abstract

**Objective:**

The microbiome is emerging as an important component of brain–body communication and may influence treatment outcomes in psychiatric disorders. This naturalistic cohort study prospectively evaluated whether baseline microbiome abundance and composition of circulating bacterial extracellular vesicles (EVs) are associated with subsequent response to selective serotonin reuptake inhibitors (SSRIs) in patients with obsessive-compulsive disorder (OCD).

**Methods:**

Thirty-two drug-naïve or medication-free patients with OCD were enrolled and assessed at baseline and at 12 weeks following the initiation of SSRI pharmacotherapy. Serum EV microbiome profiling was performed at baseline, before any pharmacological treatment was commenced, ensuring that all biological measurements reflect a pharmacologically unconfounded pre-treatment state. Good responders were defined as patients with a 35% or greater reduction in the Yale-Brown Obsessive-Compulsive Scale score at 12 weeks, while poor responders were defined as those with less than a 35% reduction. Baseline microbiome abundance and composition of circulating bacterial EVs in serum were compared between these groups.

**Results:**

Of the 32 participants, 29 completed the 12-week study. Based on the treatment response at 12 weeks, the OCD group was classified into 15 good responders (5 females) and 14 poor responders (7 females). The two groups did not differ significantly in age, sex, age of onset, baseline Y-BOCS or MADRS scores, or medication type. No significant differences in alpha or beta diversity were observed between the groups. Taxonomic analysis of pre-treatment serum EV profiles revealed a nominally higher relative abundance of the genus *Acinetobacter* in patients who subsequently showed poor response compared to good responders. However, this association did not remain statistically significant after correction for multiple comparisons, age adjustment, or compositionally-aware reanalysis.

**Conclusion:**

These results provide no statistically defensible evidence of a taxon-level microbiome difference between response groups, and the nominal *Acinetobacter* finding should not be regarded as a validated biomarker. Global diversity measures also did not distinguish the response groups. These findings are hypothesis-generating and support the feasibility of pre-treatment serum EV-associated bacterial profiling in OCD. Larger, adequately powered studies incorporating compositionally appropriate statistical methods and rigorous contamination-control procedures are warranted to determine whether reproducible microbiome–treatment response associations exist.

## 1 Introduction

Obsessive–compulsive disorder (OCD) is a chronic and debilitating psychiatric disorder characterized by recurrent, intrusive obsessions and repetitive, ritualistic compulsions (1, 2). Unfortunately, a substantial proportion of patients with OCD do not adequately respond to optimal pharmacotherapy with selective serotonin reuptake inhibitors (SSRIs) (3–5), which are considered the first-line pharmacological treatments for the disorder (6, 7). Identifying biological markers that are associated with poor treatment response could help minimize exposure to ineffective medications, reduce disease-related disability, and inform more personalized therapeutic strategies (8). Despite this clinical need, the peripheral biological signatures that distinguish eventual SSRI responders from non-responders in OCD prior to treatment initiation remain poorly characterized.

The human microbiome, defined as the collection of all microorganisms and their metabolites residing within and on the human body (9), has garnered increasing attention for its role in human health and pathology (10). Emerging evidence suggests that imbalances in the gut microbiota are involved in the pathophysiology of neuropsychiatric disorders (11–13), with the gut microbiota communicating bidirectionally with the brain through various pathways, including neurotransmitters, immune responses, and microbial metabolites (14–17). Furthermore, the microbiome has been recognized as a potential biomarker for modulating pathological conditions (18–20) and predicting treatment outcomes (21–23). Although clinical studies investigating the microbiome’s role in treatment response across neuropsychiatric disorders remain limited (16), recent evidence on depression has shown that microbiome composition can influence the response to SSRIs, with specific microbial communities associated with either favorable or diminished treatment outcomes (24, 25).

Direct evidence linking the gut microbiome to OCD pathophysiology is emerging (26). Turna et al. examined fecal microbiota in 21 medication-free OCD patients and found reduced alpha diversity, depletion of several butyrate-producing genera, and elevated plasma CRP correlated with symptom severity, suggesting a neuroimmune dimension to gut dysbiosis in OCD (27). Another study reported similar reductions in alpha diversity, along with enrichment of *Alistipes* and depletion of *Coprococcus*; these alterations persisted after treatment, suggesting trait-like microbial differences in OCD (28). More recently, fecal microbiota from drug-naïve patients with OCD induced anxiety- and compulsive-like behaviors in mice, accompanied by mPFC neuroinflammation and metabolic changes implicating succinic acid as a potential mediator (29). Collectively, these findings support a biologically plausible role for gut dysbiosis in OCD. Nevertheless, whether these microbiome alterations are associated with pharmacological treatment outcomes in OCD has not previously been examined.

A promising approach to assessing circulating microbial signals is through extracellular vesicles (EVs) - nanoscale, membrane-bound particles produced by bacteria that can enter systemic circulation, reach distal organs including the brain, and modulate immune responses and neural function (30–34). Serum-derived EVs also offer a minimally invasive window into circulating bacterial signals without requiring stool collection. In our recent study, serum EV analysis showed distinct microbial features in unmedicated patients with OCD compared with healthy controls, suggesting that altered microbiome composition and EV abundance may play a role in OCD pathogenesis (35). However, no study has yet examined whether pre-treatment EV-enriched microbiome profiles are associated with SSRI treatment response in OCD.

The present study prospectively examined the association between pre-treatment serum EV microbiome characteristics and SSRI response in patients with OCD. All participants were drug-naïve or medication-free for at least 12 weeks at serum collection, ensuring a pharmacologically unconfounded baseline state. We compared EV microbiome diversity and taxonomic composition between patients who later showed good or poor response to 12 weeks of SSRI treatment to identify candidate circulating microbial biomarkers of treatment response in OCD.

## 2 Methods and materials

### 2.1 Participants

The current cohort consisted of patients with OCD who were longitudinally recruited from a larger sample of unmedicated OCD patients in our previous cross-sectional study on the microbiome (35). The Institutional Review Boards of the Severance Hospital (Yonsei University Health System, Seoul, Republic of Korea) approved this study. All participants gave their written informed consent before participation in the study. The study was conducted in accordance with the provisions of the Helsinki Declaration (1989).

All the participants were ethnically Korean and aged between 19 and 60 years. The inclusion criteria were a current primary diagnosis of OCD, as defined by the Diagnostic and Statistical Manual of Mental Disorders, Fifth Edition (DSM-5) (36), and drug-naïve or medication-free status for at least 12 weeks prior to enrollment. Additionally, all participants had a Yale–Brown Obsessive Compulsive Scale (Y-BOCS) score of ≥18. For further details on the exclusion criteria, please refer to our previous report (35). Among 89 patients with OCD (38 women) who completed our previous cross-sectional study, 32 provided separate written informed consent to participate in the present naturalistic prospective cohort study examining the clinical response to SSRI treatment; the remaining 57 patients were not enrolled because they did not provide consent for this additional follow-up study. Recruitment for this prospective cohort took place between January 2022 and August 2023.

Serum collection for EV microbiome analysis was performed at this pre-treatment baseline, before any pharmacotherapy was initiated, ensuring that all biological measurements reflect a pharmacologically unconfounded state. At baseline, all participants were asked to complete the Y-BOCS and the Montgomery-Åsberg Depression Rating Scale (MADRS). The participants then began SSRI pharmacotherapy and were followed for a period of three months. At the 12-week follow-up, the participants completed the same assessments that were administered at baseline.

### 2.2 Measures

Symptoms of OCD and clinical responses to pharmacological treatment were assessed using the Y-BOCS (37). The Y-BOCS is a ten-item clinician-rated scale, with each item scored on a 5-point Likert scale (0–4), with higher scores indicating more severe OCD symptoms (37). In addition, depressive symptoms were assessed using the MADRS at baseline (38). The MADRS is a ten-item clinician-rated scale, with each item scored on a 7-point Likert scale (0–6), with higher scores indicating more severe depressive symptoms (38).

### 2.3 Pharmacotherapy and evaluation of SSRI treatment response

Following baseline assessment and serum collection, all patients received SSRI pharmacotherapy based on the clinical judgment of a psychiatrist who was independent of both the rater and the statistical analyst, in accordance with the Korean OCD treatment guidelines (39), which recommend SSRI monotherapy as the first-line treatment. Dosing followed routine clinical practice and was individualized rather than protocol-driven. Treatment was initiated with escitalopram 10 mg/day or fluoxetine 20 mg/day, and escitalopram was typically increased to 20 mg/day within 2 weeks if tolerated. Subsequent dose adjustments were made according to symptom response and adverse effects. Benzodiazepines were prescribed on a short-term, low-dose basis if necessary. Fluoxetine-equivalent doses (40) and lorazepam equivalent doses (41) at the 12-week follow-up are reported in **Table 1**. Medication adherence was monitored clinically by the treating psychiatrist at each visit rather than through a structured adherence instrument. No patients received formal, manualized psychotherapy such as structured cognitive behavioral therapy and exposure and response prevention during the study period; routine clinical management incorporating brief psychoeducation and supportive discussions informed by cognitive-behavioral principles was provided as part of standard medication-management visits with the treating psychiatrist, consistent with standard outpatient psychiatric practice in Korea, but was not delivered as a discrete or quantifiable intervention and could not be separately analyzed. Because serum EV microbiome profiling was completed prior to the initiation of any pharmacological treatment, neither SSRI type and dose nor benzodiazepine co-prescription could have influenced the baseline EV microbiome profiles. These treatment variables therefore represent characteristics of the post-baseline clinical course rather than potential confounders of the pre-treatment biological measurement.

**Table 1:** Baseline demographic and clinical characteristics of participants.

Variable	Good responders (n=15)	Poor responders (n=14)	*p*
Age (years)	25.1 ± 4.73	30.6 ± 8.92	0.053
Sex, male/female, n	10/5	7/7	0.462
Age of onset (years)	18.0 (15.0 ~ 23.0)	17.5 (14.0 ~ 24.0)	0.914
Y-BOCS	30.3 ± 4.20	29.0 ± 3.14	0.344
MADRS	21.3 ± 7.10	22.7 ± 8.56	0.623
Medication type			
SSRIs, escitalopram/fluoxetine, n	11/4	12/2	0.651
fluoxetine equivalent doses, mg/day	44.9 ± 5.66	45.2 ± 5.75	0.876
Concurrent Benzodiazepine, Yes/No, n	3/12	5/9	0.427
lorazepam-equivalent dose†, mg/day	0.83 ± 0.29	0.50 ± 0.00	0.197

The means ± standard deviations are provided for age, Y-BOCS, and MADRS; the median (interquartile range, IQR) is presented for age of onset.

Y-BOCS, Yale–Brown Obsessive Compulsive Scale; MADRS, Montgomery–Åsberg Depression Rating Scale; SSRIs, Selective Serotonin Reuptake Inhibitors

†Lorazepam-equivalent doses were calculated only for participants receiving benzodiazepines (good responders: n = 3; poor responders: n = 5). The SD of 0.00 in poor responders reflects identical lorazepam-equivalent values across different benzodiazepine agents. Groups did not differ significantly (Mann-Whitney U test, p = 0.197).

Treatment response to SSRIs was assessed using the Y-BOCS after 12 weeks of treatment. A good treatment response was defined as a reduction of 35% or more in the Y-BOCS score from baseline at 12 weeks (5, 42). The rater administering the 12-week Y-BOCS assessment was blinded to baseline microbiome results, and laboratory personnel performing EV isolation, sequencing, and taxonomic analysis were blinded to each participant’s treatment-response status.

### 2.4 Microbial DNA analysis

EV isolation, DNA extraction from serum, amplicon sequence analysis using EV DNA, and analysis of microbial DNA composition were described in our previous study on OCD (35). All microbial DNA analyses were conducted by *MD Healthcare*, Inc. (Seoul, Republic of Korea). For more detailed information on microbial DNA analysis, please refer to a previous report (35).

Briefly, EVs were isolated from serum by sequential centrifugation (10,000 g, 15 min, 4 °C; followed by 10,000 rpm, 10 min, 4 °C after mixing with phosphate-buffered saline) and subsequent 0.22-μm filtration to remove residual cells and debris. EV-associated bacterial DNA was extracted after boiling isolated EVs (100 °C, 40 min) and a further centrifugation step (18,312 g, 30 min, 4 °C) to remove residual particulate matter, using a DNeasy PowerSoil Pro Kit (QIAGEN), and quantified with the QIAxpert system. Bacterial genomic DNA was amplified by PCR targeting the V3–V4 hypervariable region of the 16S ribosomal RNA gene, and sequencing libraries were prepared and sequenced on the Illumina MiSeq platform. Paired-end reads were processed in QIIME2 (version 2021.4) using DADA2 for quality filtering, denoising, and chimeric-read removal, and amplicon sequence variants were taxonomically classified with a naïve Bayes classifier trained on the SILVA 138 reference database; reads classified as chloroplast or mitochondrial sequences were excluded. All samples from both response groups were processed and sequenced in the same batches on the same day to minimize batch effects, and all wet-laboratory procedures were performed under standard contamination-control measures (gowning, masking, and glove use) within a dedicated clean-sample area, with post-PCR library preparation carried out in a physically separate room from DNA extraction to minimize amplicon carry-over. Dedicated negative extraction controls (e.g., no-template blanks) were not processed in parallel with patient samples in the present study; because serum represents a low-microbial-biomass matrix that is susceptible to reagent- and environment-derived contamination, this is acknowledged as a limitation below. Full methodological details, including primer sequences and DADA2 quality-filtering parameters, have been reported previously (35).

### 2.5 Statistical analysis

Data normality was assessed using the Shapiro-Wilk test. For normally distributed data, results were presented as mean ± standard deviation. Non-normally distributed data were presented as median and interquartile range (IQR). Between-group comparisons between good and poor responders were conducted using independent t-tests for normally distributed continuous variables and the Mann–Whitney U tests for non-normally distributed continuous variables. Categorical variables were analyzed using Fisher’s exact test.

**Figure 1:**
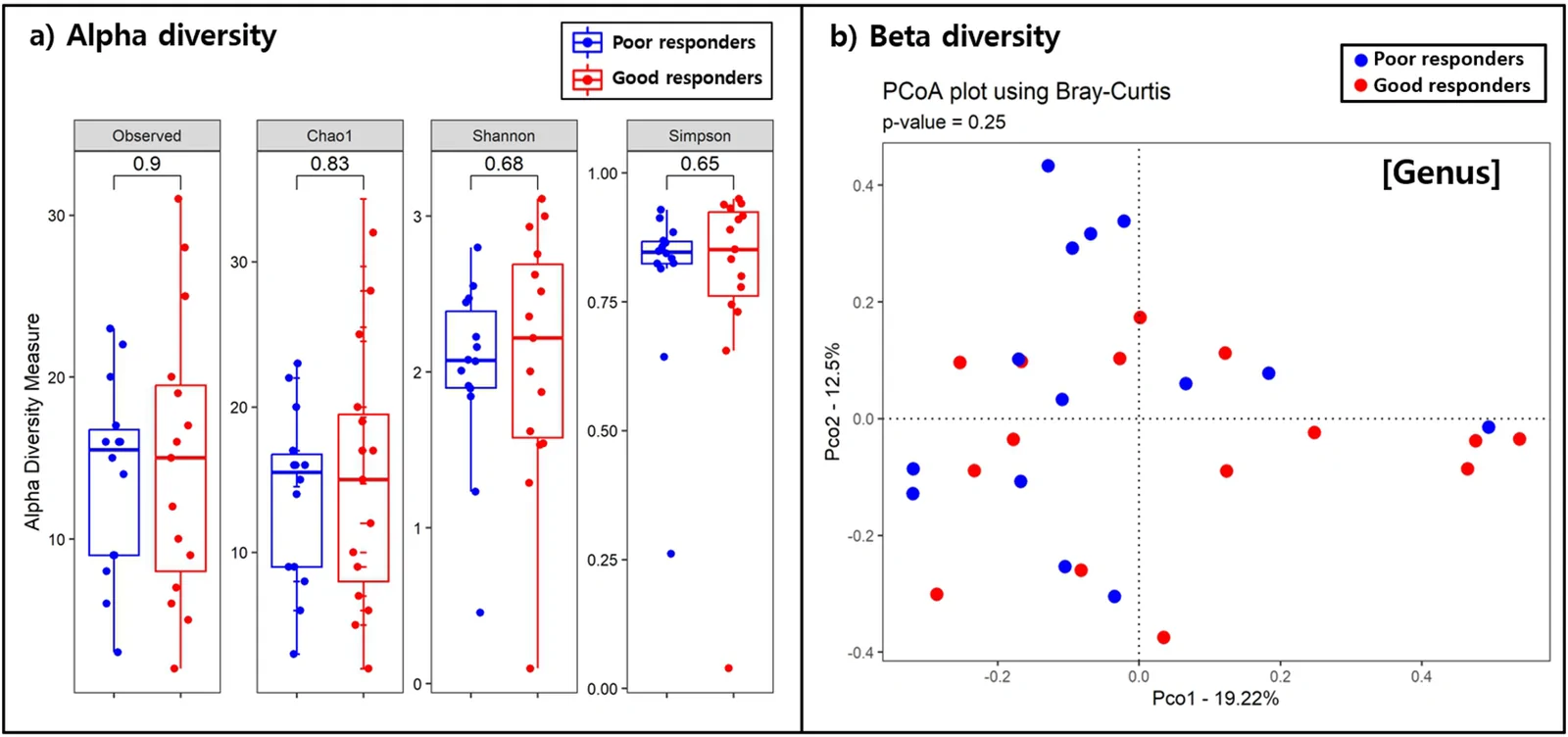
**(A)** Box plots of alpha diversity metrics, including observed ASVs, Chao1, Shannon, and Simpson indices, stratified by treatment response type (poor responders in blue and good responders in red). **(B)** Principal coordinate analysis (PCoA) of Bray-Curtis dissimilarity at the genus level. Poor responders are in blue, while good responders are in red. The plot is based on the first two principal components (PCo1 and PCo2), with the distance between points reflecting compositional differences between samples.

Alpha diversity, which refers to the richness and evenness of microbial taxa within individual samples (43), was evaluated using several metrics, including observed amplicon sequence variants (ASVs), Chao-1 index, Shannon index, and Simpson index. Beta diversity, which refers to the compositional differences in microbial communities between samples (44), was assessed across all taxonomic levels (phylum, class, order, family, genus, and species). The PCoA plot presented in **Figure 1B** is based on genus-level Bray-Curtis dissimilarity, as this level provides an informative balance between taxonomic resolution and biological interpretability.

All statistical tests were two-sided, and statistical significance was set at an alpha level of 0.05. Given the exploratory nature of the study and the limited sample size, genus-level taxonomic comparisons between good and poor responders were not adjusted for multiple comparisons; the implications of this approach, including a genus-wide false discovery rate correction, are discussed in the Discussion/Limitations. *Post hoc* sensitivity analyses, including Quade’s nonparametric analysis of covariance and rank-based partial correlation, were performed; the rationale for these analyses and the selected covariate are reported alongside the corresponding results. All analyses were performed using IBM SPSS Statistics for Windows, version 28.0 (IBM Corp., Armonk, NY, USA) and R version 4.4.2 (R Foundation for Statistical Computing).

## 3 Results

Of the 32 participants initially enrolled, three were lost to follow-up during the 3-month pharmacotherapy period due to withdrawal of consent or missed visits, and their treatment response could not be assessed, leaving 29 patients with OCD who completed the 12-week study. All patients at follow-up were taking SSRIs, comprising escitalopram (n = 23) and fluoxetine (n = 6), and some were taking concomitant medications, including benzodiazepines (n = 8). According to the treatment response types at 12 weeks, the OCD group was classified into 15 good responders (five females) and 14 poor responders (seven females). No significant differences were found between good and poor responders with respect to age, sex, age of onset, baseline scores of OC and depressive symptoms, and medication types (**Table 1**). Age was the only variable showing a trend-level imbalance between groups (*p* = 0.053), whereas other variables were balanced. Accordingly, age was included as a covariate in *post hoc* sensitivity analyses to assess whether this imbalance could account for the taxonomic finding.

Alpha diversity did not differ significantly between good and poor responders across any of the four indices examined (Observed *p* = 0.90; Chao1 *p* = 0.83; Shannon *p* = 0.68; Simpson *p* = 0.65; **Figure 1A**). Beta diversity, assessed using the Bray-Curtis dissimilarity matrix, showed no significant between-group differences at any taxonomic level examined (phylum, class, order, family, genus, or species). A principal coordinate analysis (PCoA) plot based on the Bray-Curtis dissimilarity matrix at the genus level is presented for visualization purposes (*p* = 0.25; **Figure 1B**).

**Figure 2:**
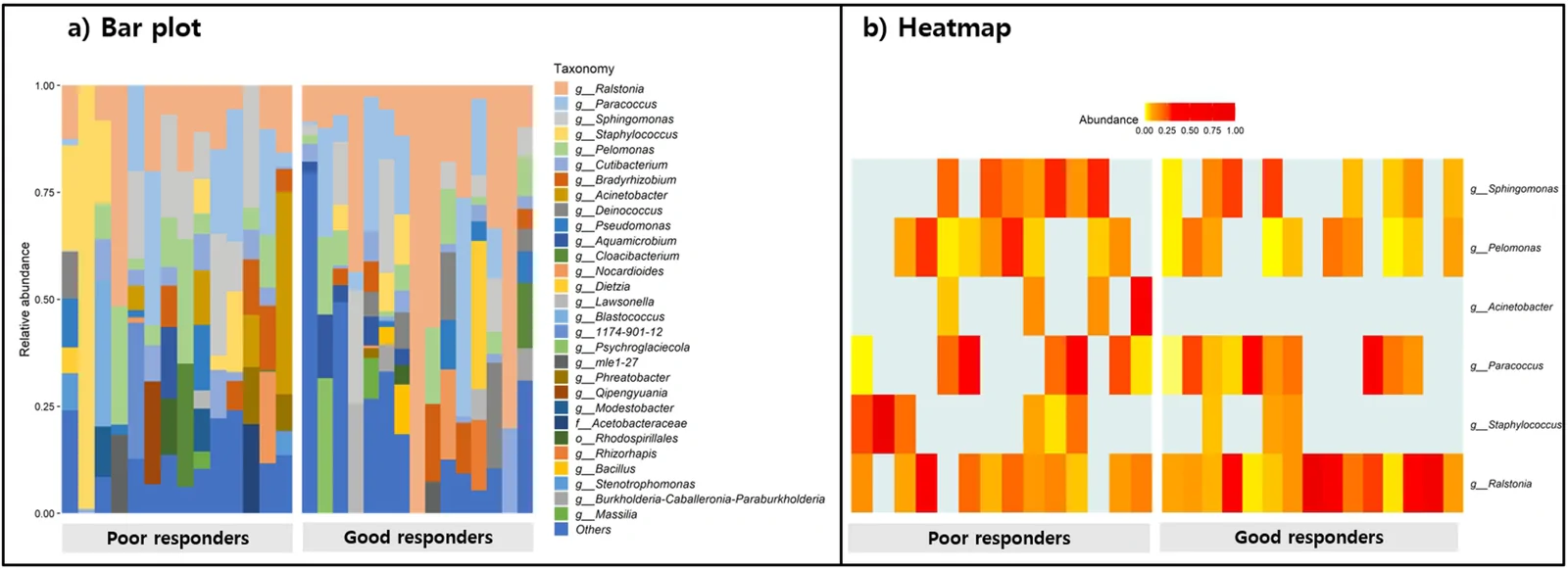
**(A)** Bar plot showing the relative abundance of circulating bacterial extracellular vesicles at the genus level at baseline (pre-treatment), stratified by 12-week SSRI treatment response, among OCD patients (N = 29). **(B)** Heatmap illustrating the differences in the relative abundance of the serum microbiome at the genus level at baseline (pre-treatment), categorized by treatment response type.

Taxonomic analysis of microbiome composition revealed that the most abundant phyla in both groups were *Proteobacteria, Actinobacteria, Firmicutes, Bacteroidetes*, and *Deinococcota*. No significant between-group differences were observed at the phylum, class, order, or family levels. At the genus level, however, the pre-treatment relative abundance of *Acinetobacter* was significantly higher in patients who subsequently showed poor response than in good responders after 12 weeks of SSRI treatment (Mann-Whitney U = 75.00, standardized test statistic = - 2.183, *p* = 0.029, rank-biserial r = 0.29). In a sensitivity analysis addressing the trend-level age imbalance between groups noted above (*p* = 0.053), a Quade nonparametric analysis of covariance with age as a covariate showed no statistically significant between-group difference in *Acinetobacter* abundance (F (1,27) = 3.116, *p* = 0.089). The relative abundance of the serum microbiome at the genus level by treatment response is shown in **Figure 2**.

**Figure 3:**
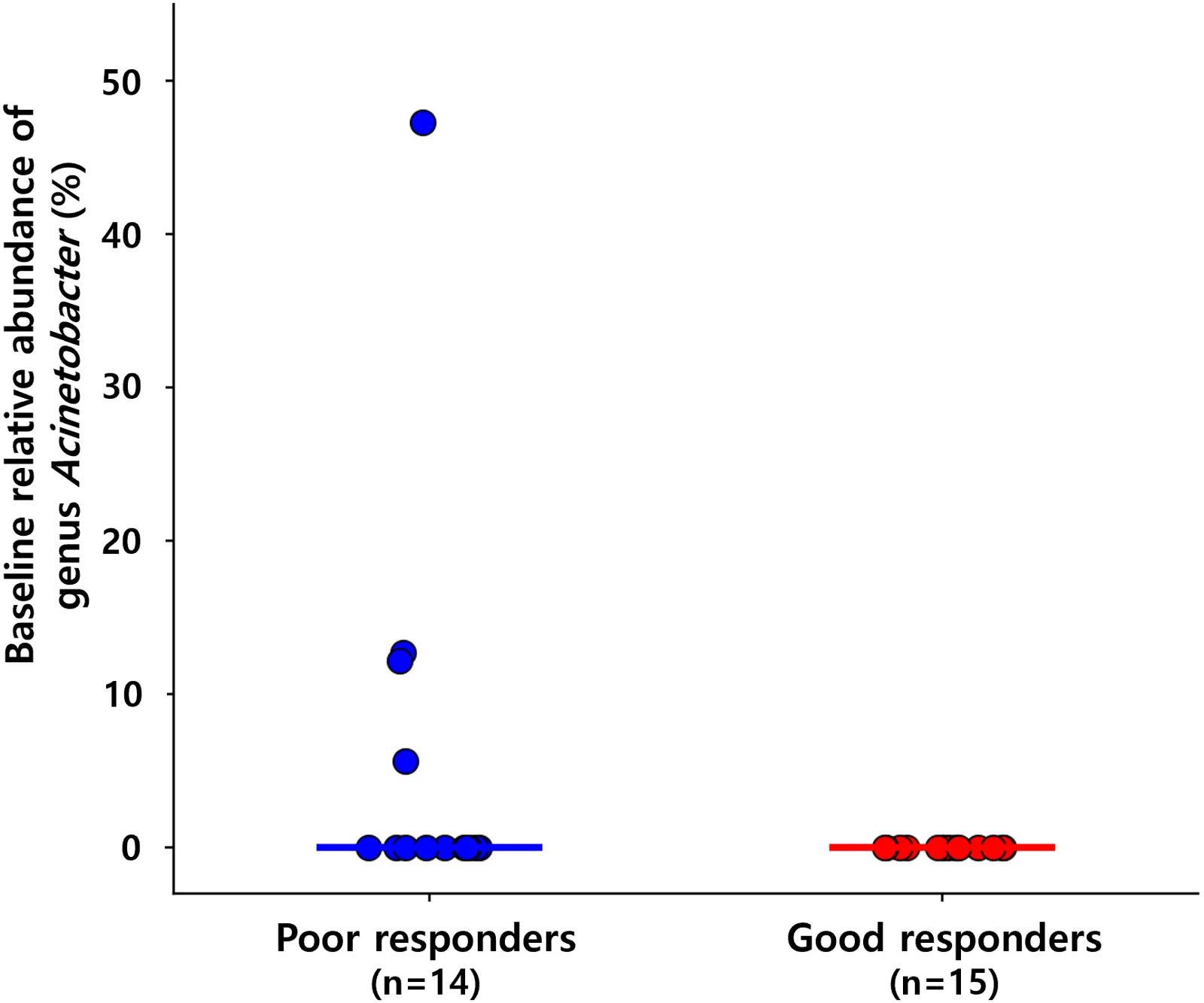
Individual participant-level values for baseline relative abundance of the genus *Acinetobacter*, stratified by subsequent SSRI treatment response at 12 weeks (poor responders, n = 14; good responders, n = 15). Horizontal bars indicate group medians. *Acinetobacter* was detectable in 4 of 14 poor responders and 0 of 15 good responders (Fisher’s exact test for detection status, p = 0.042).

Inspection of individual-level data (**Figure 3**) showed that *Acinetobacter* was detectable in only 4 of 29 participants, all of whom were poor responders (4/14), whereas none of the good responders showed detectable levels (0/15). An exploratory Fisher’s exact test comparing detection (presence vs. absence) between groups was nominally significant (*p* = 0.042).

As a complementary continuous analysis, baseline *Acinetobacter* abundance was inversely correlated with percentage reduction in Y-BOCS at 12 weeks across all participants (Spearman’s rho = - 0.42, *p* = 0.022). This association attenuated to a trend level after adjusting for age (rank-based partial *r* = - 0.34, *p* = 0.067), consistent with age partially, but not fully, accounting for this association.

To further evaluate this finding using compositionally-aware methods appropriate for relative abundance data, we conducted a reanalysis using the ALDEx2 framework (Monte Carlo Dirichlet resampling with Jeffreys-prior centered log-ratio [CLR] transformation, 500 instances) applied to the full genus-level count table (102 taxa detected in at least one participant). Under this approach, the *Acinetobacter* association was not statistically significant (median p = 0.47 across Monte Carlo instances; only 5.2% of instances reached p < 0.05), with a negligible effect size (median CLR-scale Cohen’s d = 0.13).

## 4 Discussion

The present prospective study examined the potential role of pre-treatment microbiome features derived from systemically circulating bacterial EVs in relation to subsequent SSRI treatment response in patients with OCD. A key methodological strength of this study is its pre-treatment design: serum EV microbiome profiling was performed at baseline in drug-naïve or medication-free patients before any pharmacotherapy was initiated, ensuring that the observed microbiome profiles reflect a pharmacologically unconfounded biological state. Our findings revealed that the relative abundance of *Acinetobacter* was nominally higher in patients who subsequently showed poor response to SSRI treatment compared to good responders. These findings tentatively suggest that pre-treatment EV-enriched microbiome profiles - specifically, elevated circulating *Acinetobacter* abundance - should not be regarded as a robust or reproducible candidate biomarker of poor SSRI treatment response in OCD without further validation. However, as detailed below, this association did not remain significant after adjustment for baseline age, after correction for multiple comparisons, or in a compositionally-aware reanalysis, and should be interpreted with substantial caution. It is important to note that this study evaluated group-level associations rather than predictive accuracy; measures such as sensitivity, specificity, or other indices of predictive validity were not assessed, and the term “candidate biomarker” is used here to denote a correlate warranting further investigation rather than a validated predictive tool ready for clinical application.

While an increasing body of evidence highlights the involvement of microbiota in neuropsychiatric disorders (14, 45–47), including OCD (27–29), no prior study has investigated the association between microbiome features and pharmacological treatment outcomes in this patient population. This overrepresentation of *Acinetobacter* in poor responders is particularly noteworthy. *Acinetobacter* is a highly diverse and complex genus commonly found in the environment (48, 49) and some *Acinetobacter* species have been previously associated with inflammatory responses and impaired immune regulation (50–52). Considering the ability of microbial EVs to modulate systemic inflammation and immune responses (53–55), elevated circulating *Acinetobacter*-associated signals in poor responders may reflect an inflammatory or immune-dysregulatory state that impairs SSRI efficacy.

Although the specific mechanisms underlying the association between pre-treatment *Acinetobacter* abundance and subsequent treatment response remain to be established, several biologically plausible pathways warrant consideration. First, elevated *Acinetobacter* abundance may contribute to a persistent low-grade inflammatory state, potentially impairing SSRI efficacy. Certain *Acinetobacter* species have been reported to induce inflammatory responses (50, 51) or to promote intestinal inflammation and disintegration (56). Chronic low-grade inflammation and alterations in the immune system have been implicated in the pathophysiology and persistence of several psychiatric disorders (57–59), including OCD (60, 61). Second, *Acinetobacter* may interact with gut-brain axis signaling pathways, potentially altering serotonin synthesis, metabolism, or receptor sensitivity and thereby modulating the therapeutic effects of SSRIs (15, 62). Third, and more speculatively, SSRIs themselves have been reported to exhibit direct antimicrobial activity against *Acinetobacter* species *in vitro*, acting through efflux pump inhibition, induction of oxidative stress, and disruption of bacterial membrane integrity (63). Although the *in vitro* antimicrobial concentrations substantially exceed therapeutic plasma levels, this observation raises the speculative possibility that elevated *Acinetobacter* abundance in poor responders may partly reflect reduced susceptibility to SSRI-mediated antimicrobial effects - a hypothesis that requires direct experimental investigation. It must be emphasized that all proposed pathways represent speculative interpretations grounded in the existing literature and cannot be inferred directly from the present data. In particular, the inflammatory hypothesis discussed above is not supported by any inflammatory biomarker data (e.g., CRP, cytokines) collected in this study; no such markers were measured, and this mechanistic discussion should therefore be read as motivation for future research rather than as an explanation grounded in our findings.

However, this association attenuated to a trend level after adjustment for age (Quade nonparametric analysis of covariance; F (1,27) = 3.116, *p* = 0.089), suggesting that the unadjusted association may be partly attributable to age. This interpretation is supported by a weak-to-moderate positive correlation between age and *Acinetobacter* abundance across the full sample (Spearman’s rho = 0.32, *p* = 0.09), consistent with age acting as a potential confounder. In addition, inspection of individual-level data suggests that *Acinetobacter* was undetectable in most participants, and that all four participants with detectable abundance were poor responders. This pattern suggests that the association may be better characterized as a difference in the probability of detectable colonization rather than a graded shift in abundance. A similar attenuation was observed for the continuous association between Acinetobacter abundance and Y-BOCS reduction after adjusting for age (partial r = - 0.34, *p* = 0.067). A compositionally-aware reanalysis using the ALDEx2 framework (Monte Carlo Dirichlet resampling with centered log-ratio transformation across all 102 detected genus-level taxa) likewise found no significant *Acinetobacter* association (median p = 0.47; only 5.2% of Monte Carlo instances reached significance), with a negligible effect size, further reinforcing that this signal does not withstand statistical approaches appropriate for compositional microbiome data. Taken together, these sensitivity analyses indicate that the *Acinetobacter* finding should be interpreted as preliminary and hypothesis-generating rather than as a robust or confirmed association.

Our analysis revealed no significant differences in the overall microbial diversity, including both alpha and beta diversity, between good and poor responders as classified at 12 weeks of SSRI treatment. This pattern is consistent with findings from prior human microbiome studies in OCD, in which alpha diversity differences between patients and controls were modest or failed to survive multiple comparison correction (27, 28), and suggests that broad diversity measures may not be the most sensitive indicators of treatment-relevant microbiome differences. Evidence from other conditions, such as inflammatory bowel disease (64) and depression (65), has shown that overall microbial richness and evenness often fail to consistently correlate with clinical outcomes. Instead, the presence or absence of specific microbial taxa, along with their neuroactive metabolites, showed stronger associations with clinical features (47). These findings underscore the importance of taxon-specific analyses when investigating microbiome-treatment response relationships in psychiatric disorders. However, the null findings for alpha and beta diversity should be interpreted with caution in the context of the present sample size, as they may partly reflect limited statistical power rather than a true absence of effect.

The present study had several limitations. First, serum is a low-microbial-biomass matrix and dedicated negative extraction controls were not processed; thus, reagent- or environment-derived contamination—particularly given that *Acinetobacter* is a known contaminant in low-biomass 16S studies—cannot be excluded. Because contamination may be more readily detected in samples with lower endogenous biomass, a systematic difference in contamination susceptibility between groups cannot be ruled out. Sample-level biomass (e.g., 16S copy number) was not quantified, and formal characterization of the isolated fraction (e.g., nanoparticle tracking analysis, transmission electron microscopy, or EV marker Western blotting) was not performed, so we cannot confirm that the signal was exclusively EV-encapsulated. Second, the small sample size (N = 29) may have limited power to detect small-to-moderate effects; null findings for alpha and beta diversity should be interpreted cautiously. Selection bias is also possible, as baseline characteristics of non-participants could not be compared. Third, the *Acinetobacter* finding was nominally significant only and did not survive false discovery rate correction (q = 0.483); age showed a nominal between-group imbalance (p = 0.053), and full multivariable adjustment was not feasible given the sample size (risk of overfitting with only 14 poor responders); age was the only imbalanced covariate, while medication type was determined only after baseline sampling. Fourth, medication dosing was naturalistic, adherence and psychoeducation were not quantified, and dietary (66), lifestyle data (67, 68), and other microbiome-relevant covariates (e.g., antibiotic/probiotic use, gastrointestinal disorders, smoking, body mass index) were not systematically collected. Fifth, the mechanism linking pre-treatment *Acinetobacter* abundance to treatment response remains unknown. Finally, serum EV-enriched microbial profiles reflect circulating bacterial EV-associated signals rather than gut microbiome composition; paired stool and serum EV sampling in longitudinal designs would clarify the gut–systemic microbiome relationship during OCD treatment.

In summary, this study did not identify a robust, statistically defensible taxon-level microbiome difference between good and poor SSRI responders: the nominal *Acinetobacter* finding did not survive correction for multiple comparisons, adjustment for age, or a compositionally-aware reanalysis, and should not be regarded as a validated biomarker. Global measures of microbial diversity likewise did not distinguish responders from non-responders. These findings are hypothesis-generating and demonstrate the feasibility of pre-treatment serum EV-enriched microbiome profiling in OCD. Larger, adequately powered studies incorporating compositionally appropriate statistical methods, rigorous contamination controls, and formal EV characterization are warranted to determine whether reproducible microbiome-treatment response associations exist and to inform future microbiome-related research in OCD.

## Data Availability

The raw data supporting the conclusions of this article will be made available by the authors, without undue reservation.
